# Lattice-tip dual energy ablation creates effective ventricular lesions through transmural scar that might inadvertently increase lead capture thresholds on the opposing surface

**DOI:** 10.1007/s10840-026-02356-0

**Published:** 2026-05-22

**Authors:** Vishal Luther, Laura Brodie, Justin Chiong, Peter Calvert, Daniel Walker, Dhiraj Gupta, Nathan Denham

**Affiliations:** 1 Liverpool Heart & Chest Hospital NHS Foundation Trust, Thomas Drive, Liverpool, L14 3PE UK; 2 Liverpool Centre for Cardiovascular Science, University of Liverpool, Liverpool, L7 8TX UK; 3 Medtronic, Medtronic LTD, Watford, UK

**Keywords:** Ventricular tachycardia, Scar, Lattice, Ablation, Radiofrequency, Pulsed field, Tissue depth, Device lead threshold

## Abstract

**Background:**

Achieving adequate lesion depth during ventricular tachycardia (VT) ablation within scar is challenging with conventional radiofrequency energy (RF). The lattice-tip catheter enables dual-energy ablation (RF & pulsed field [PF]) with enhanced tissue penetration.

**Objective:**

We report on 3 cardiac implantable electronic device (CIED) patients with transmural apical left ventricular (LV) scars and drug-refractory VT. Each underwent endocardial LV ablation using RF & stacked PF. Ablation included delivery near the opposing surface of CIED leads, at sites critical for the VT circuit. CIED parameters were assessed pre- and post-procedure, as well as at interval follow-up.

**Results:**

VT was eliminated with complete non-inducibility achieved in all cases, and there has been no recurrence at early follow-up (4-6months). However, each case demonstrated inadvertent CIED lead capture threshold rise (apical right ventricular x2, anterolateral LV coronary sinus branch x1), with partial recovery over time. Findings correlated with ablation near opposing lead electrodes.

**Conclusion:**

Dual-energy lattice-tip ablation creates effective ventricular lesions through transmural scar that may increase CIED capture thresholds on the opposing myocardial surface. Our report recommends vigilance during ablation opposing CIED leads, balancing the respective wall thickness and potential for lead damage against the gravity of the arrhythmic presentation.

## Introduction

The success of endocardial left ventricular (LV) ablation through transmural scar is often dependent on the delivery of energy into the intramural or subepicardial space where critical ventricular tachycardia (VT) circuit components may lie. Radiofrequency (RF) ablation with conventional solid-tip catheters may lack sufficient penetration through scar, limiting ablation efficacy [1]. Preclinical experience with the Sphere-9 lattice-tip catheter (Medtronic, Minneapolis, MN) suggests it generates deeper ventricular lesions through scar, using a combination of higher power radio-frequency (RF) energy with larger surface area [2], as well as the ability to deliver repetitive monopolar pulsed field (PF) ablation in the same location [3–5]. Early clinical experience has demonstrated efficacy during VT ablation [6, 7], but some concerns with altered cardiac implantable electronic device (CIED) behaviour [8]. Herein we describe 3 cases of dual energy lattice-tip endocardial LV ablation involving patients with apical transmural scars, successfully eliminating the VT substrate, but resulting in inadvertent CIED lead threshold rise on the opposing surface.

## General approach

Our centre first used the Sphere-9 lattice-tip catheter for VT ablation in November 2025. The system has been preferred in patients with CIEDs presenting with either failed conventional RF ablation, or in patients presenting with VT storm or recurrent hospitalisation despite class I/III anti-arrhythmics. Procedures were performed under general anaesthesia and with intracardiac echocardiography (ICE) (AcuNav, Siemens Medical Solutions). CIEDs were interrogated pre-procedure to assess baseline thresholds. The LV was accessed transeptally. Substrate maps were collected with the Sphere-9 and the accompanying Affera electro-anatomical mapping system (Medtronic, Minneapolis, MN). Voltage and activation time were derived from closed unipolar electrograms (EGM) based on peak-peak voltage and maximal negative unipolar derivative respectively. Scar was empirically defined at 0.5mV, though dynamically adjusted during cases, as per our previous work using legacy mapping systems [9]. Baseline programmed ventricular stimulation was performed and activation maps were collected for haemodynamically tolerated VT.

**Table 1:** Case summary

	LV scar	Device	Lead issue	Ablation proximity	Pre-ablation	Post-ablation	~ 1 month	~ 4 months
Case 1	Apical HCM	Medtronic Evera dual chamber ICD	RV lead: Sprint Quattro^TM^ single coil 6935M	Opposing	0.75V @ 0.4ms	2.375V @ 1ms	2V @ 0.4ms	1.625V @ 0.4ms
Case 2	LAD infarct	Medtronic Claria CRT-D	RV lead: Sprint Quattro^TM^ single coil 6935M	In proximity, not opposing	1V @ 0.4ms	1.25V @ 1ms	1.5V @ 0.4ms	
Case 3	LAD infarct	Medtronic Claria CRT-D	LV lead: Medtronic Attain Stability^TM^ Quad 4798	Opposing	1V @ 0.4ms (LV1-LV2) Undocumented threshold for LV 2, 3, 4)	4V @ 1ms (LV1-LV2) >6.00V @ 1ms LV 2, 3, & 4	3.5V @ 1ms (LV1-LV2) >6.00V @ 1ms LV 2, 3, & 4	2.5V @ 1ms (LV1-RV coil)

Ablation targeted diastolic potentials in VT, aiming to achieve VT termination where possible, as well as local abnormal ventricular activities (LAVA) and late potentials (LP) in sinus/paced rhythm, manually tagged on the map. An increase in local impedance between the mini-electrodes was used as a surrogate for contact and confirmed with ICE. Given the risk of transient local EGM abolition with PF, RF was delivered first (HexaGen generator, Medtronic), temperature limited through nine surface thermocouples. A standard ventricular preset was used (60 °C target, 40% current output limit, irrigation rate of 30 mL/min) with each lesion limited to 30 s. RF ablations that exceeded an electrode temperature > 50 °C were tagged as 12 mm red disks.

Monopolar PF applications (HexaPulse generator, Medtronic; 1500 pulses per train, 12 trains per application, inter-train delay of 350ms, duration ~ 5.5 s per application) were selectively delivered at sites of VT termination with RF, as well as good pace-map matches to any documented VT, or in areas of incomplete LAVA elimination with RF alone. PF applications were stacked, up-to 4 times, to maximize energy penetration through scar [3–5]. The locations of PF ablation that exceeded an electrode temperature > 2 °C above baseline were tagged as 7 mm green disks.

Complete non-inducibility using triple extra-stimuli to refractoriness was the targeted procedural endpoint, as well as abolishment of LAVA to achieve electrical isolation of the scarred segment. All patients had remote transmitters for subsequent ICD downloads. All patients consented to their anonymised data being shared towards publication, and host institutional approval was obtained for this evaluation.

## Case 1

A 62-year-old male with septal hypertrophic cardiomyopathy and secondary apical aneurysm was repeatedly hospitalised with monomorphic VT (150 bpm) refractory to oral Quinidine and Nebivolol (prior thyrotoxicosis on Amiodarone). He had a Medtronic Evera dual chamber ICD (Sprint Quattro™ single coil 6935 M defibrillator lead). Cardiac MRI measured apical wall thickness of ~5 mm. The 12 lead ECG suggested an apical-septal exit.

**Fig. 1:**
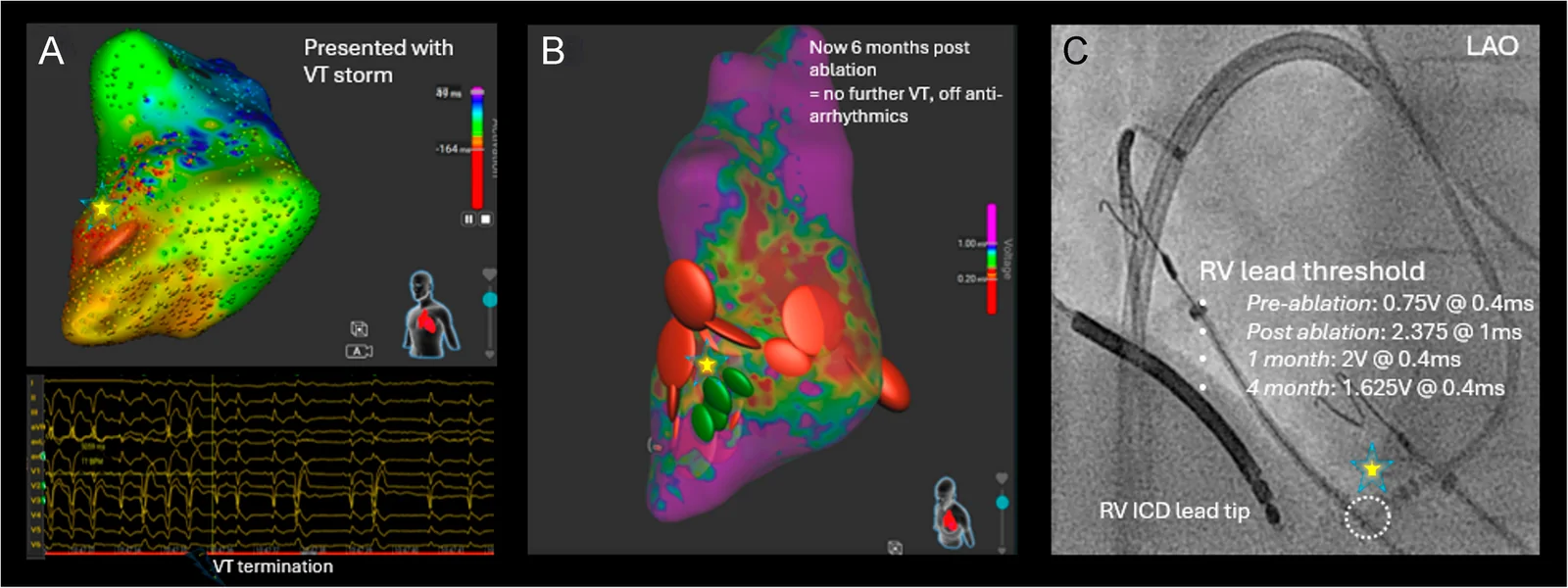
**A** activation map in VT revealed a broad breakout on the LV endocardial apical septal wall. A single RF lesion (red disk) terminated VT after ~10secs. **B** substate voltage map is displayed in modified LAO, with voltage settings between 0.2-1.0mV. Tissue voltages <0.2mV are coloured red. The location of RF (red) and PF (green) ablation disks are included. **C** saved fluorogram of the Sphere 9 (circled white) approximating to the site of VT termination in LAO, and the corresponding location of the RV ICD lead tip. A yellow star is seen on all 3 images to estimate the site of VT termination

The patient presented to the lab in incessant VT. The activation map (9299 points) revealed a broad breakout on the endocardial apical septal wall, suggesting a deeper intramural circuit (Fig. 1A). Mapping time was 19 min, which included acquiring the entire LV geometry and activation data in VT, navigating the lattice carefully through the neck of the hypertrophied septum into the apical aneurysm, and confirming endocardial contact with dimensions of the aneurysm using intracardiac echo. A single RF lesion resulted in delayed termination of VT after ~ 10–20 s at this site. A full 30 s RF lesion was delivered, and VT was no longer seen. Stacked PF applications were colocalised, and a cluster of RF and PF applications were delivered around this region to consolidate. The remaining LAVA and LP were ablated using RF (total 23 applications, average temp 53 °C, max temp 59 °C, impedance drop 7%) and repetitive PF (total 18 applications) in critical areas (Fig. 1B). Total ablation time was 12 min. Total procedural time was 178 min without complication. Quinidine was stopped, and there has been no VT recurrence after ~ 6 months.

The pre-ablation RV lead bipolar capture threshold was 0.75 V @ 0.4ms. A post procedure acute increase in RV threshold to 2.375 V @ 1ms was noted, without change in sensing or impedance, suggesting transmural LV ablation towards the distal ICD electrode. A fluorogram demonstrating an approximation between the lattice and the RV lead tip during ablation was fortuitously saved during the procedure (Fig. 1C). The threshold reduced during follow-up: 1 month post ablation to 2 V @ 0.4ms and 4 months to 1.625 V @ 0.4ms.

## Case 2

**Fig. 2:**
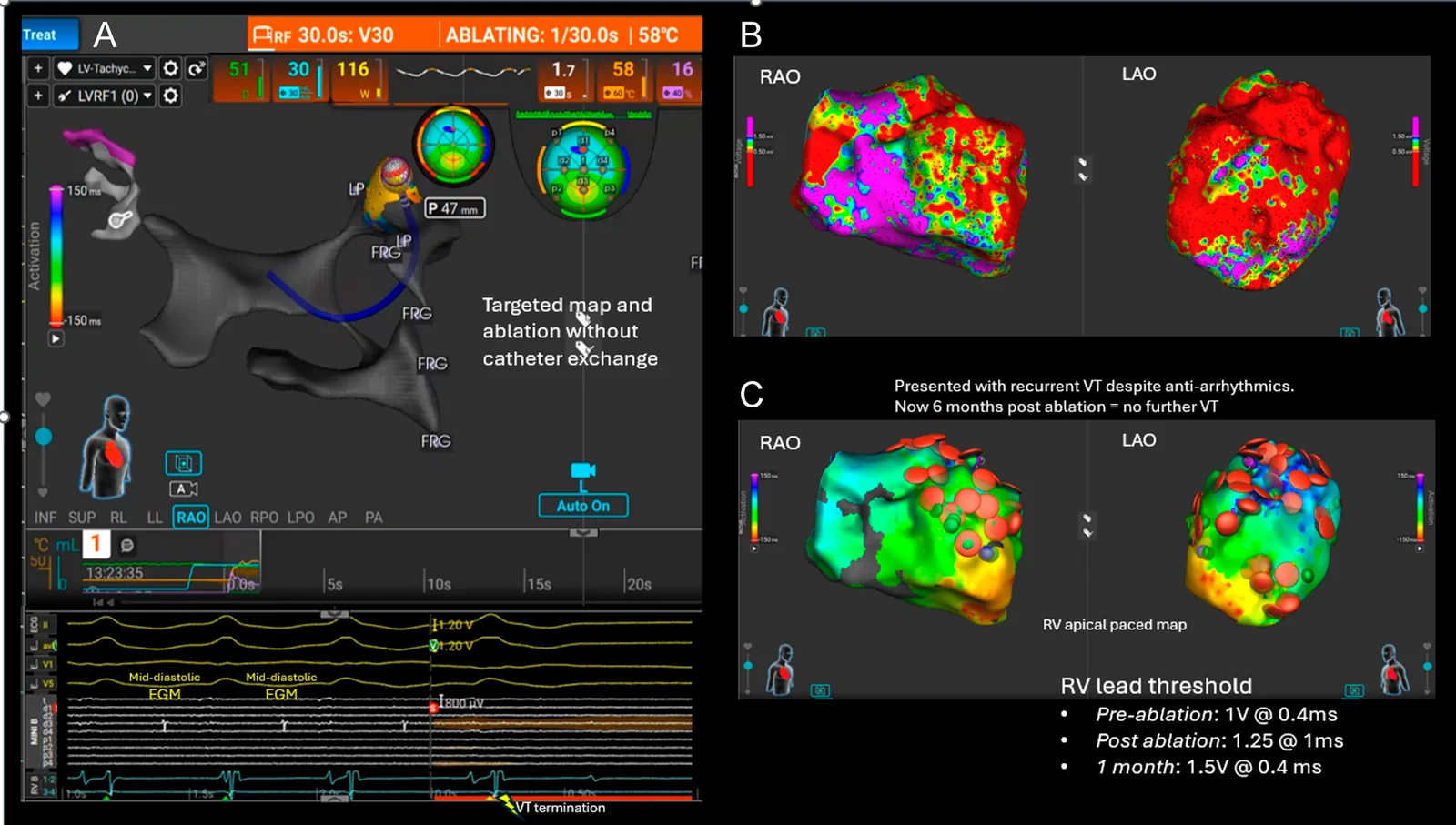
**A** the Sphere 9 is seen in RAO, retroflexed to the apical anterior LV surface, identifying mid-diastolic EGMs (bottom) in VT. RF ablation was delivered immediately, without collecting an activation map, resulting in VT termination within 1 second (top orange bar). **B** substate voltage map is displayed in RAO and LAO, with voltage settings between 0.5-1.5mV. Tissue voltages <0.5mV are coloured red. **C** the corresponding substrate activation map, collected in an RV apical paced rhythm, reveals the site of earliest LV activation in red, and its proximity to the RF (red) and PF (green) ablation disks is provided

A 73-year-old male with remote LAD infarct presented with repeated hospitalisation for VT despite chronic Amiodarone therapy. He had a Medtronic Claria CRT-D device (Sprint Quattro™ single coil 6935 M defibrillator lead) with an LV lead positioned down a posterior CS branch, distant from the infarct site. Anti-tachycardia pacing (ATP) was ineffective. Transthoracic imaging revealed an akinetic and aneurysmal LV mid-apical cavity. The 12 lead ECG in VT suggested an apical-lateral exit. Catheter manipulation within the infarcted substrate induced VT, which demonstrated mid-diastolic local activation in the mid-apical anterolateral surface. Immediate RF ablation (without performing activation mapping or catheter exchange) terminated VT within 1 s (Fig. 2A).

Substrate mapping (6763 points) confirmed extensive apical low voltage tissue < 0.5mV (Fig. 2B). LAVA and LP were ablated using a combination of 30 s RF (total 43 applications, average temp 54 °C, max temp 64 °C, mean impedance drop 6.5%), and stacked PF (total 30 applications) (Fig. 2C). The total ablation time was 22 min following which VT was non-inducible. Procedural duration was 151 min and without complication. There has been no VT recurrence after ~ 6 months.

The pre-ablation RV lead bipolar capture threshold was 1 V @ 0.4ms. A post procedure acute increase in RV threshold to 1.25 V @ 1ms was noted, without change in sensing or impedance. At 1 month post ablation this had reduced to 1.5 V @ 0.4ms suggesting some ablation induced oedema towards the distal ICD electrode. The displayed LV activation map (Fig. 2C) was collected in an RV apical paced rhythm with the site of earliest LV breakout coloured red - some ablation disks are in the region of the site of earliest breakout, but not overlapping.

## Case 3

A 61-year-old male with remote LAD infarct and prior endocardial LV mid-septal ablation (3 years earlier) presented in ICD shock storm despite Amiodarone. Cardiac MRI revealed akinetic and aneurysmal LV mid-apical cavity with wall thickness of 4 mm. He had a Medtronic Claria CRT-D device (Sprint Quattro™ single coil 6935 defibrillator lead) with an LV lead (Medtronic Attain Stability™ Quad 4798) situated down an anterolateral LV branch of the CS. VT was resistant to ATP. The 12 lead ECG suggested an apical-anterior exit.

**Fig. 3:**
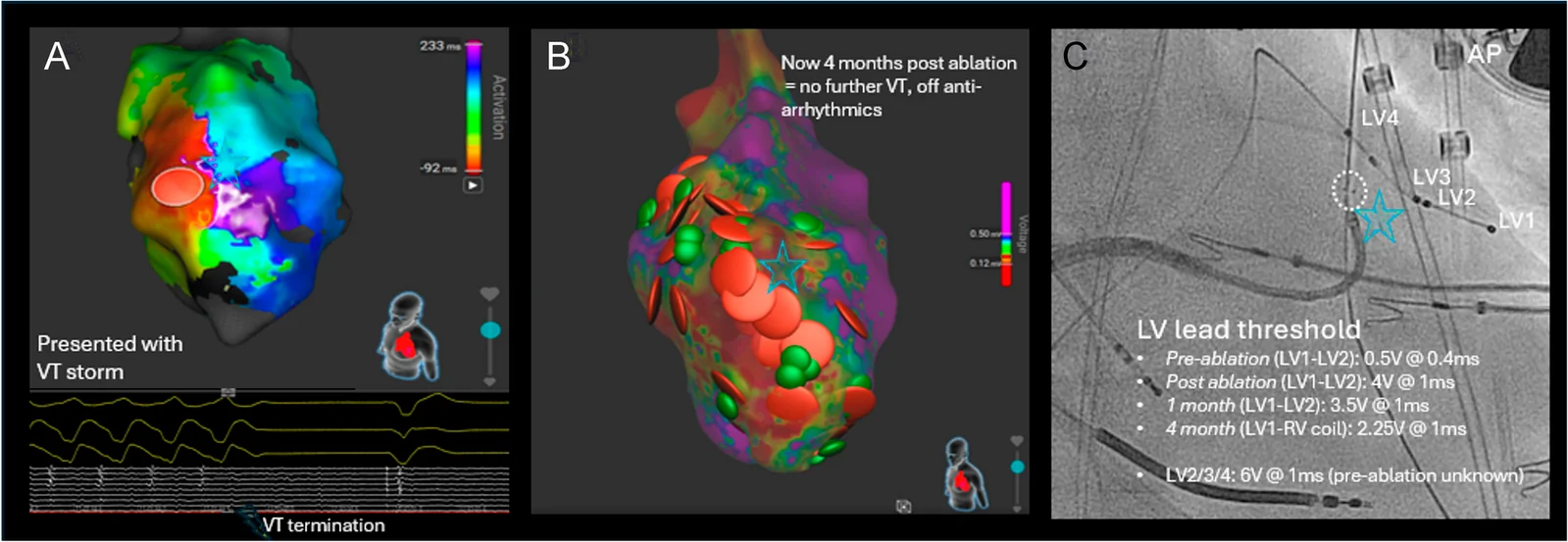
**A** activation map of VT4 revealed a figure-of-8 re-entry with critical isthmus in the apical antero-lateral LV surface. RF ablation within the exit (red) terminated VT. **B** substate voltage map is displayed in modified LAO with voltage settings between 0.12-0.5mV. Tissue voltages <0.12mV are coloured red. The location of RF (red) and PF (green) ablation disks are included. **C** saved fluoroscopic image of the Sphere 9 (circled white) approximating to the site of VT termination in AP, and the corresponding location of the LV quadripolar lead. A yellow star is seen on all 3 images to estimate the site of VT termination

3 different VT morphologies were seen during mapping, exiting the border zone of a large mid-apical scar (basal lateral, apical lateral and basal septal). RF ablation (with subsequent stacked PF) in areas of pre-systolic activation rapidly terminated VT to sinus rhythm on each occasion prior to the need for external cardioversion. A 4th haemodynamically tolerated VT remained inducible, and activation mapping revealed figure-of-8 re-entry with a critical isthmus in the apical anterior-lateral aspect of the scar (Fig. 3A). This site was within proximity of the opposing epicardial LV pacing electrode. RF ablation within the isthmus terminated this VT, and stacked PF applications were consolidated. A total of 48 RF applications were delivered (mean temp 53 °C, peak temp 63 °C, impendence drop 4%), and a further 62 stacked PF applications were delivered, over a total ablation time of 27 min (Fig. 3B), to eliminate LAVA and LP. Given our experience of ICD lead threshold rise seen in case 1 and 2, we limited RF ablation opposing the ICD lead-tip to 15-seconds with no PF applications. The total procedural time was 177 min, addressing 4 VTs, without complication and with complete non-inducibility at procedural end. Amiodarone was stopped, and there has been no VT recurrence ~ 4 months post-ablation.

Post procedure, no change in RV lead threshold was observed. However, the pre-ablation LV lead capture threshold of 1 V @ 0.4ms, measured from LV1-LV2, increased post procedure to 4 V @1ms, with a fall in pacing impedance (722 to 437 Ohms), suggesting transmural LV ablation towards the epicardial antero-lateral surface. Furthermore, no myocardial capture was apparent when measured from the more proximal poles (i.e. LV2, LV3, LV4 - (threshold > 6.00 V @ 1ms)), though measurements pre-ablation were not documented for comparison. A fluorogram demonstrating an approximation between the lattice and the more proximal poles of the LV quadripolar lead during ablation was fortuitously saved during the procedure (Fig. 3C). There was minor improvement in threshold 2 months post ablation (LV1-LV2 capture threshold 3.5 V @ 1ms, 494 Ohms), with no capture on the proximal poles. Polarity was reconfigured to LV1-RV coil, with capture threshold 2.5 V @ 1ms at month 4. No noise has been detected on the local LV EGM during follow-up.

Table 1 presents a summary of the temporal changes in pacing threshold for cases 1–3.

## Discussion

We describe our early experience using the Sphere 9 dual energy lattice-tip ablation catheter in 3 patients with apical left ventricular transmural scars (wall thickness ~5 mm) presenting in extremis with drug refractory VT and recurrent hospitalisation or storm. Using a protocol of 30-second RF, as well as repetitive PF at critical VT components, aiming to eliminate all LAVA and late potentials, complete non-inducibility was achieved, without VT recurrence at early follow-up.

The lattice-tip catheter represents a significant advancement in efficiency and effectiveness for VT ablation. The ability to integrate high‐density mapping and dual energy ablation, all without catheter exchange, is ideally suited to VT ablation within scar, especially relevant in comorbid patients, at risk of haemodynamic compromise with prolonged procedures. Our learning curve was short, with procedural duration less than 3 h in each case, with frequent VT termination without investing time mapping the entire VT circuit (Fig. 2A). We limited our RF lesions to 30 s, as longer lesions have been associated with steam pop [10], and instead, stacked up to 4 PF repetitions to gain better penetrance through scar in areas of importance (e.g. sites of VT termination), as has been applied previously [6, 7, 11]. We observed no occurrences of steam pop with our RF applications.

In creating effective lesions, we observed an inadvertent increase in CIED lead capture thresholds situated on the opposing myocardial surface. There have already been safety concerns of energy-induced CIED injury with both RF and PF delivered from the lattice-tip catheter. This includes inadvertent VF induction with RF in close proximity to ICD leads [12–14]; permanent damage to an ICD generator with PF in proximity of a proximal high voltage coil in the superior vena cava [15] and similar damage after left septal VT ablation [16] without direct contact with the ICD system. Whilst these concerns were unexpected and under investigation, ours might be considered somewhat predictable when ablating towards the opposing surface of device lead tips, especially in patients with relatively thin-walled apical scars as seen in our 3 cases. Conventional RF ablation with solid-tip catheters is already known to risk CIED lead threshold rise [8], and pre-clinical studies from dual energy lattice ablation have determined lesion depth >5 mm [2, 4, 5], exceeding the measured wall thickness in our 3 patients, perhaps more likely risking injury to opposing CIED leads. Amplified current density near the CIED lead tip may also increase local heating and injure surrounding tissue [8].

Having learned from the first 2 cases, we used a combination of fluoroscopy and ICE to judge proximity to the RV ICD in case 3 and reduced ablation near the lead tip, with no resultant ICD lead issues. As dual-energy ablation technologies become increasingly used, vigilance may be required with ablation opposing device lead tips, balancing the wall thickness and potential for lead damage against the gravity of the ventricular arrhythmia. Given the severity of the arrhythmic presentation in our 3 patients, an increase in pacing capture thresholds without compromising lead integrity was an acceptable sacrifice. Notably, the thresholds in our 3 patients have improved somewhat with time.

## Limitations

Given these lead threshold rises were only identified after the procedure, the precise distance between the ablation catheter electrode and the CIED electrode was not measured in any of the 3 cases, hence cannot be accurately provided. As our ablation protocol comprised both RF and PF, neither one can be considered more responsible based on these cases alone, rather the additive effect of greater scar penetrance with dual energy lattice ablation. Post-ablation imaging (e.g. contrast CT or MRI) has not been undertaken to assess local lesion effects. We hope that our cases stimulate prospective studies to address these unknowns.

## Data Availability

The data that support the findings of this study are not openly available due to reasons of sensitivity and are available from the corresponding author upon reasonable request. Data are located in controlled access data storage at Liverpool Heart and Chest Hospital NHS Foundation Trust.
